# 3D bioprinting matrices with controlled pore structure and release function guide in vitro self-organization of sweat gland

**DOI:** 10.1038/srep34410

**Published:** 2016-10-03

**Authors:** Nanbo Liu, Sha Huang, Bin Yao, Jiangfan Xie, Xu Wu, Xiaobing Fu

**Affiliations:** 1 Key Laboratory of Tissue Repair and Regeneration of PLA, and Beijing Key Research Laboratory of Skin Injury, Repair and Regeneration, First Hospital Affiliated to General Hospital of PLA, Beijing 100048, P. R. China; 2 Department of Thoracic and Cardiovascular Surgery/Huiqiao Medical Center, Nanfang Hospital, Southern Medical University, Guangzhou, 510515, P. R. China; 3 Institute of Basic Medical Sciences, General Hospital of PLA, Beijing 100853, P. R. China; 4 School of Medicine, Nankai University, Tianjin, 300071, P. R. China

## Abstract

3D bioprinting matrices are novel platforms for tissue regeneration. Tissue self-organization is a critical process during regeneration that implies the features of organogenesis. However, it is not clear from the current evidences whether 3D printed construct plays a role in guiding tissue self-organization *in vitro*. Based on our previous study, we bioprinted a 3D matrix as the restrictive niche for direct sweat gland differentiation of epidermal progenitors by different pore structure (300-μm or 400-μm nozzle diameters printed) and reported a long-term gradual transition of differentiated cells into glandular morphogenesis occurs within the 3D construct *in vitro*. At the initial 14-day culture, an accelerated cell differentiation was achieved with inductive cues released along with gelatin reduction. After protein release completed, the 3D construct guide the self-organized formation of sweat gland tissues, which is similar to that of the natural developmental process. However, glandular morphogenesis was only observed in 300-μm–printed constructs. In the absence of 3D architectural support, glandular morphogenesis was not occurred. This striking finding made us to identify a previously unknown role of the 3D-printed structure in glandular tissue regeneration, and this self-organizing strategy can be applied to forming other tissues *in vitro*.

Sweat gland regeneration is an ongoing clinical challenge in cutaneous wound repair. Without effective restitution of lost sweat gland cells during wound healing, heat intolerance can occur in survivors of large-scale deep burns, which seriously affect their quality of life^1^. Unlike other glandular tissue, sweat gland morphogenesis *in vitro* had never been reported before because they have low regenerative potential even in response to injury^2^. A promising solution to tissue regeneration could be establishment of a 3D printed extracellular matrix (ECM) because a controlled environment can play an important role in guiding cell and tissue level functions including directing tissue-specific stem cell specification and influencing tissue development^3^. In our final goal of clinical practice, 3D bioprinting is especially advantageous because it can integrate multiple biophysical and biochemical cues spatially for cellular regulation and ensure highly ordered and complex structures with great stability and reproducibility^4^.

The perfect regenerative strategy might rely on guiding tissue morphogenesis at the cell level. Although the process of 3D bioprinting in medical science is remarkable, *in vitro* self-organization of tissue structures in 3D constructs has not been fully proved. Our recent study demonstrated the feasibility of using 3D bioprinting to enhance the specific differentiation of epithelial progenitors (EPs)^5^, the further question is whether or not 3D printed architecture is responsible to guiding sweat gland morphogenesis. Recently, the influence of physical parameters such as pore size and geometry is of rising interest in the field of 3D printing techniques^6^,^7^,^8^, yet the role of them in cell to glandular tissue organization remains unexplored. To address this problem, we collected the entire dynamic events after 3D bioprinting *in vitro* and focused on defining the role of the architectural impact during the developmental process, which may offer new insights into the significance of 3D printed constructs in the promotion of tissue regeneration.

In this study, 3D porous structure is a key point to promote glandular ingrowth by providing suitable space. We developed 3D bioprinting matrices with an accurate pore structure by using a controlled printing parameter. Additionally, with cell printing, the gelatin-based hydrogel allows for producing an inductive niche with defined ECM components^9^,^10^ and the opportunity to introduce predefined porosity for sweat gland reconstruction in 3D constructs. Furthermore, controlled release of the inductive factors at target time points could ensure the specific differentiation of epidermal lineages^11^. A potent gland-lineage inductive factor for EPs involves plantar dermis (PD), dermal components in mouse plantar extraction that are a collection of protein molecules, with bone morphogenic protein (BMP-4) the predominant growth-factor protein^12^,^13^,^14^,^15^. Since the relationship between EP behavior and the inductive niche, including factors and ECM-mimetic biomaterials^5^,^16^, have been well investigated, we emphasize on attending the architectural impact on cell differentiation associated with tissue morphogenesis using 2 size of printing nozzles by which the different structure can be manipulated.

Here we investigated embedded EP differentiation and PD delivery in response to different porous constructs, and potential sweat gland self-organized formation within 3D constructs. We used well-controlled fabrication and precise observing approaches to firstly identify the role of 3D printed constructs in guiding glandular tissue regeneration *in vitro* and optimize this strategy for sweat gland regeneration. Further, results of this investigation might have implications for guiding other tissue morphogenesis and propelling 3D bioprinting application in regenerative medicine.

## Results

### Characteristics of 3D constructs

**Figure 1:**
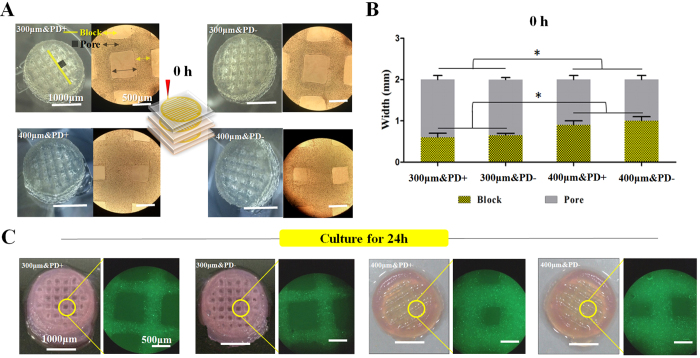
Characteristics of printed porous constructs. (**A**) Two typical architectures of the porous construct, block and pore, identified right after printing. (**B**) Width of block and pore between 300- and 400-μm–printed constructs at 0 hr. Data are mean ± SD. *P < 0.05. (**C**) Embedded cell distribution and structural stability of printed constructs after 24-hr culture. (scale bar: 1000 μm for macroscopic images; 500 μm for microscopic images).

By direct-writing with the pneumatic 3D bioprinting system, 2 cell-laden bioink types (with and without PD) plus 2 printing nozzles (300 and 400 μm) were paired for rapid fabrication. With the layer-by-layer printing process, crisscross blocks and square pores (top to bottom) were determined as 2 typical architectures and could be measured on microscopy (Fig. 1A). The 300-μm–printed constructs produced larger pore width than 400-μm–printed constructs (1.4 ± 0.1 vs 1.1 ± 0.1 mm), but the block width was smaller (0.6 ± 0.1 vs 1.1 ± 0.1 mm) (Fig. 1B). Four representative porous constructs (20 × 20 × 5 mm) maintained structural stability and cells were homogeneously embedded after 24-hr culture (Fig. 1C).

### Cell viability and proliferation

**Figure 2:**
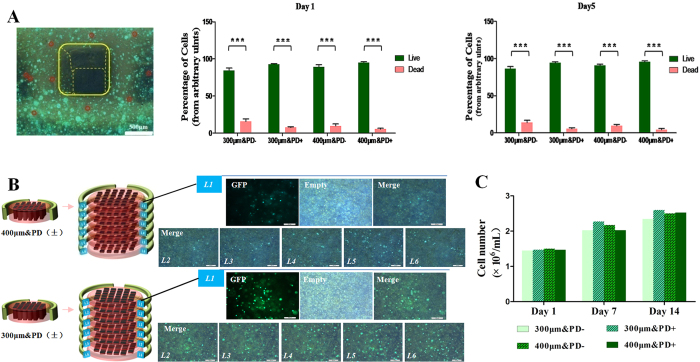
Cell viability in 3D printed constructs. (**A**) Live-dead cell counting at day 1 and 5 of culture. Green dots are live cells and dark, circled dots are dead cells (scale bar, 200 μm). Data are mean ± SD. ***P < 0.001. (**B**) Cell distribution on every layer of scaffolds. Insets show 300-μm group with a greater density than 400 μm. (**C**) Cell number at day 1, 7, and 14 of culture (P > 0.05). Data are mean ± SD.

Most embedded cells in all groups retained GFP-positive expression, and a small number of GFP-negative cells (red-enclosed) were also noted (Fig. 2A). These dead cells could be linked to shearing caused by the printing extrusion. The 300- and 400-μm–printed constructs did not confer differences in cell viability. Compared with day 1, at day 5, we observed no abnormal cell death in the 3D culture. With the construct-split observation method, we calculated the overall cell amount of each construct at the target time, with cell density and cell distribution demonstrated in Fig. 2B. Cell density was greater in the 300- than 400-μm–printed construct, which could be directly linked to the narrower block architecture. Cells proliferated, as seen by an increased amount of embedded live cells during 3D culture (Fig. 2C). Similarly, constructs did not differ in cell number at each day of culture, which suggested that both 300- and 400-μm nozzles were biocompatible in the printing system.

### Gelatin reduction and BMP-4 release

**Figure 3:**
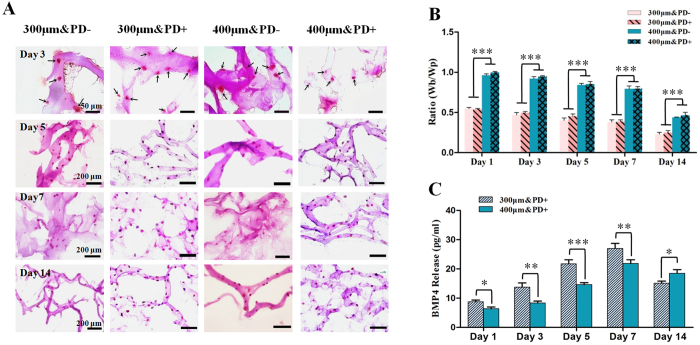
Biomaterial degradation analysis and bone morphogenic protein 4 (BMP-4) release. (**A,B**) Biomaterial degradation of scaffolds with culture. (**A**) Histology and (**B**) ratio (Wb/Wp) of 3D-printed scaffolds at day 1, 3, 5, 7, and 14 of culture (scale bar: 50 μm for day 3; 200 μm for day 5, 7 and 14, respectively; arrow: embedded cell). (**C**) BMP-4 release from 3D-printed scaffolds over time. Data are mean ± SD. *P < 0.05, **P < 0.01, ***P < 0.001.

Because of the cross-link reaction of sodium alginates with calcium chloride, the structures were relatively fixed in 3D culture, thereby providing attachment sites for embedded cells. However, because gelatin in the hydrogel is responsible for the release function, the reduction changes of gelatin must be quantified. The dynamic changes by HE staining suggested that gelatin progressively vanished with time and completely disappeared on day 14 (Fig. 3A). As the width of the block became narrower, the pore width expanded, for corresponding decrease in structure-related ratio (Fig. 3B).

BMP-4 content was prolonged and gradually released along with gelatin reduction during week 1 but was less with 400- than 300-μm–printed constructs (Fig. 2C). This increasing trend was related to progressive gelatin dissolution and degradation. Thus, the bioprinted 3D constructs we describe are suitable for protein delivery for cell differentiation.

### Induced sweat gland cell differentiation and morphogenesis

**Figure 4:**
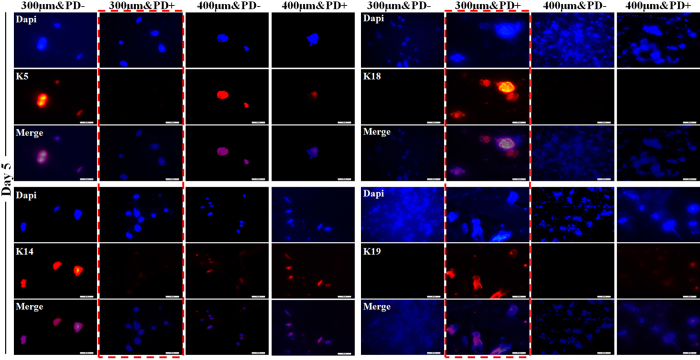
Epidermal progenitor cells embedded in 3D-printed constructs differentiation into sweat gland cells. Immunostaining assay at day 5 of culture with K18, K19, K5, K14 for sweat gland cell differentiation of epidermal progenitors. All markers are red and Dapi staining of nuclei is blue. (scale bar, 50 μm) Data are mean ± SD. *P < 0.05.

**Figure 5:**
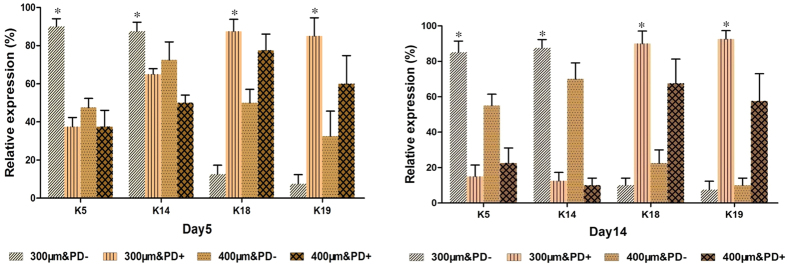
The comparison of relative marker expression quantified by ImageJ. Immunostaining assay at day 5 and day 14 of culture with K18, K19, K5, K14 for sweat gland cell differentiation of epidermal progenitors. At least 3 samples were scored in 3 independent replicates for each group and at least 100 cells were scored for each sample. Data are mean ± SD. *P < 0.05.

EPs routinely maintain the main epidermal markers K5 and K14 (representing primary creatine kinases of stratified epithelia). Once the cells differentiate into sweat gland cells, they should lose original epithelial markers and gain luminal epithelial markers (e.g., K18, CK19). Therefore, we used the conversion of marker expression as a differentiation indicator. Notably, EPs in the 300 μm&PD+ construct showed strong expression of K18 and K19 as compared with other constructs at day 5 (Fig. 4). After the initial 14-day culture, a more prominent conversion of marker expression was achieved in the 300 μm&PD+ construct. These trends were well consistent with the results of inductive factor releasing along with gelatin reduction (Fig. 5).

**Figure 6:**
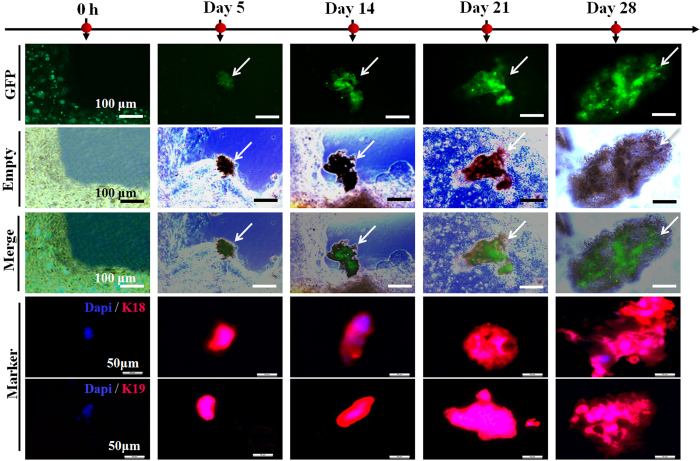
The progress of sweat gland morphogenesis in random samples of 300 μm&PD+ –printed construct. (scale bar: 100 μm for fluorescence images; 50 μm for immunofluorescence images).

**Figure 7:**
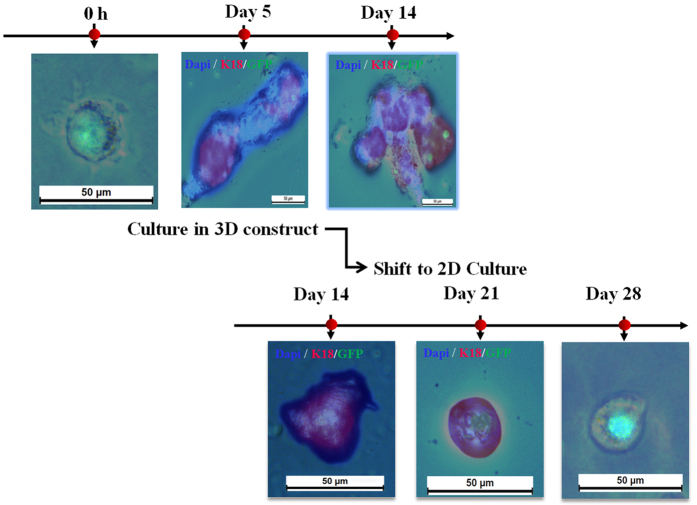
The changes of cell differentiation and morphogenesis in random samples of 300 μm&PD+ –printed construct when shift to 2D culture at day 14 of culture. Epidermal progenitor cells embedded in 3D-printed constructs differentiation into sweat gland cells by immunostaining assay at day 5 of culture with K18 and aggregation into sweat gland-like structure at day 14 of culture. All markers are red and Dapi staining of nuclei is blue. After shifting to 2D culture, the structure disappearance at day 21 of culture and differentiated cells decrease at day 28 of culture (scale bar, 50 μm).

To assess the potential for glandular morphogenesis, we measured the long-term culture of each group. The morphology of sweat gland tissue was only detected in the 300 μm&PD+ construct after day 28 of culture (Fig. 6). In contrast, differentiated cells in 400-μm–printed constructs (regardless of PD delivery) and 300 μm-μm–printed constructs (without PD delivery) were gradually decreased. There might not be enough space to complete the cell aggregates, or differentiated cells could fail to aggregate if not enough number is available. The 300-μm–printed constructs conferred a significant increase in sweat gland cell aggregates from weeks 1 to 2, and the specific sweat gland-bud emerged at week 3. Then these buds develops into sweat gland tissues, which is similar to that of the natural developmental process. However, once deprivation of 3D architectural support, the self-organized formative process was gradually terminated (Fig. 7).

## Discussion

Bioprinted cell matrices are an emerging strategy to address the clinical need for cell therapy and regenerative medicine^4^,^16^. The ability of 3D constructs to ensure embedded cell-specific differentiation is of great use in sweat gland regeneration. Besides the controlled-release gland lineage-inductive effects, porous architecture has a profound effect on promoting the sweat gland self-organization formation. Our current findings reveal the previously unknown role of 3D-printed structure in spatial controls over the differentiated cell behavior in glandular tissue developmental stages by an *in vitro* culture model. These findings might significantly deepen the understanding of 3D bioprinting technology.

The adapted printing parameter for the resulting fabrication and postprocessing that we used can fulfill all requirements for 3D bioprinting for regenerative medicine. We found that these constructs ensured long-term stability with architectural wholeness and the release of inductive cues by biodegradation. 3D porous constructs printed with a 300-μm nozzle diameter produced the best gland lineage-differentiation and tissue morphogenesis *in vitro* as compared with constructs produced with a 400-μm nozzle. By combining the benefits of suitable pore structure and release function during the printing process, 3D constructs had a synergistic promotional effect on producing an inductive niche for sweat gland regeneration.

The 2 types of modified 3D printed constructs did not differ in cell viability, although the expression of sweat gland markers was slightly higher with 300- than 400-μm–printed constructs on day 5. Thus, accurately controlled structural characteristics guarantee the rational behavior of cells cultured in constructs, and a larger pore size might have stronger ability to induce differentiation. Strikingly, our results showed sweat gland self-organization *in vitro* that 300-μm constructs with PD which guided the process of bud emergence and glandular branching growth after long-term culture, while few or no sweat gland cells was detected in 400-μm–printed constructs after 14 days. These constructs with a 1.4-mm pore size may benefit differentiated cell aggregation and help them more easily gather around connecting pores for assembly process.

Besides architectural support, the delivery of material may be of greater importance for cell differentiation toward a specific sweat gland lineage. In the present study, PD released from constructs and executing the inductive effects led to a significant increase in number of differentiating cells in a relatively short period. This promotional effect on induction may be attributed to at least in part, improved release function by degradation of modified construct. Gelatin and sodium alginate forms hybrid cross-linked hydrogels through the cross-linking of sodium alginate^17^. The factor released from the hydrolytically degradable gelatin in the hybrid scaffolds might have met the local inductive cue demand for EPs for their survival and specific differentiation. During this period, evaluation of the release properties of the different 3D printed constructs revealed that these constructs offer a modest and synchronous release in 2 weeks. When release finished, the constructs continually acted the guiding role in tissue morphogenesis *in vitro* by mimicking the native microenvironment, which is consistent with the results from transplantation *in vivo*.

Various researchers have proposed that porous biodegradable matrices obtained with 3D printing play an important role in cell function^3^,^18^,^19^. Conventional techniques used to manufacture scaffolds lack full control of the pore morphology and architecture as well as reproducibility^20^,^21^,^22^. In this study, our printing strategy both controlled printing parameter and achieved high cell viability. Another important role of 3D printing is directing sweat-gland differentiation by including living EPs and inductive factors simultaneously during fabrication without the limitations of time and culture conditions. Notably, the significant differences was observed between cell long-term culture in 2D and 3D conditions. These findings further underline the importance of 3D architecture in highly efficient differentiation of stem cells. In particular, the present study is the first report that *in vitro* glandular tissue morphological formation can be promoted by 3D-printed constructs. The extent and mechanisms to which these response of cells to pore geometries explain our results clearly require further elucidation.

Our findings represent a first proof of glandular cell to tissue-level organization occurred within the 3D bioprinted construct *in vitro* and pave the way for further understanding into a previously unknown role of the 3D-printed porous architecture in glandular tissue developmental stage. This self-organizing strategy might opens up the intriguing possibility of regenerating other tissues by *in vitro* microenvironmental manipulations of specific stem cell fate, which would be of particular interest for clinical applications of 3D bioprinting technology. Furthermore, the data from this study may help achieve the reproducibility and standardization in 3D bioprinting for regenerative medicine.

## Methods

### Cell isolation and culture

All animal experiments were carried out in accordance with the guidelines of the Institutional Animal Care and Use Committee of Chinese PLA General Hospital (Beijing, China). All experimental protocols were approved by the Institutional Animal Care and Use Committee of Chinese PLA General Hospital (Beijing, China). EP isolation was as previously described^5^. Briefly, EP was collected from dorsal skin of E12.5 embryonic mice [C57BL/6-Tg (ACTB-EGFP)10sb/J, Jackson Laboratory] and cultured with 2 ml phosphate buffered saline (PBS, pH = 7.4) containing 10% penicillin-streptomycin, then divested of subcutaneous fat. Then, the skin was minced into 1-mm^3^ pieces and digested with 2 mg/ml Dispase II (Sigma), 2 mg/ml collagenase I and 0.25% Trypsin-EDTA for 30 min. Digestion of trypsin was terminated by neutralization with medium containing 10% fetal bovine serum. Finally, absolute EP was harvested and incubated with Dulbecco’s modified Eagle’s medium [Nutrient Mixture F-12 (Ham) (1:1) D-MEM/F-12] (Gibco) supplemented with 10% fetal bovine serum (Gibco), 1% penicillin-streptomycin (Gibco). Cultures at passages 2 to 4 with density 6 × 10^5^ cells/ml were used.

### Bioink preparation

Bioink was prepared as described^5^ and modified by using an optimization formulation. Briefly, cells and PD were embedded into hydrogels by using a 48-ml hyperthermia-dissolved solution of 0.2 g/ml gelatin (Sigma) mixed with a 24-ml solution including 0.04 g/ml sodium alginates (Sigma) at 25 °C. All used vats, vat-paired inner sealers, and nozzles were autoclaved and maintained aseptic at 4 °C before use. The whole assembling bioink process was strictly sterilized.

### 3D Porous construct fabrication

The prepared bioink was divided into 4 equal parts and transferred into sterile vats with different nozzle diameters (300 and 400 μm); 2 parts were randomly selected as the PD+ printing group. Each printing vat was loaded with 2 ml EPs and 1 ml PD. The other 2 parts were each loaded with 2 ml EPs and 1 ml PBS. Therefore, 4 groups were created, 300 μm&PD+ and -PD- and 400 μm&PD+ and -PD-, before bioprinting. The synthetic bioink of all groups was maintained in an identical sterilized chamber at 4 °C to keep it gelated.

From our previous printing procedure, the bioprinting was performed as follows. First, the printing platform (Regenovo 3D bioprinter, China) with a temporal-fixed 100-mm Petri dish used as a substrate was rapidly cooled for 30 min to 4 °C and underwent ultraviolet sterilization. As the process progressed, square pores were generally formed in the constructs through the layer-by-layer rotation of the meandering thread pattern. Finally, each fresh finished construct was immediately sprinkled with 1 ml sterile 10% calcium chloride for 10 min to crosslink sodium alginate, then immersed with the sweat gland cell (SGC) medium (50% DMEM and 50% F12 supplemented with 5% fetal calf serum [FCS] (Gibco), 10 ng/ml epidermal growth factor [EGF] (Sigma), 2 ng/ml liothyronine sodium (Gibco), 0.4 μg/ml hydrocortisone succinate (Gibco), 1 ml/100 ml insulin-transferrin-selenium [ITS] (Gibco), 1 ml/100 ml penicillin-streptomycin solution) and cultured in the incubator at 37 °C in a humidified atmosphere of 5% CO_2_.

### Cell distribution and viability

We used an improved practical *in situ* microscope-based method for observing fluorescent cells. For convenience, we split the construct into 6 transversal layers from upside down according to different imaging focal lengths, labeled L1 to L6. Then, for all images collected from each layer in a single visual field, we classified the cells with green fluorescence as “live cells” and the ones losing fluorescence as “dead cells”. Finally, we calculated and added the overall amount of live and dead cells from L1 to L6. Three samples were analyzed for each group. At least 3 samples were scored in 3 independent replicates for live/dead calculation.

### Construct degradation and BMP-4 release

To study the degradation of printed constructs in different groups, *in vitro*-cultured samples were used after 1, 3, 5, 7, and 14 d of culture. To acquire proper cross-sectional images, randomly selected constructs were first fixed with 4% paraformaldehyde overnight at 4 °C, then paraffinized, embedded, sectioned at 8 μm with use of a standard microtome, and pasted onto clean glass slides for hematoxylin and eosin (HE) staining and imaged under a light microscope (Olympus BX-41, Japan).

Similarly, improved *in situ* microscopy method was used to evaluate construct degradation in a large scale. Specifically, for each construct we first measured its linear block width (μm) and pore width (μm) at different times. Next, we defined an architecture-related ratio (Wb/Wp) indirectly linked to the degradation of the bioprinted construct in a large scale, where Wb is the width of the block and Wp the width of the pore. Finally, we analyzed the change in ratio over time to describe the construct degradation.

BMP-4 concentration in the medium was measured by ELISA (ABIN1568654; Antibodiesonline.cn, Aachen, Germany). At least 3 samples were scored in 3 independent replicates for BMP-4 release experiments.

### *In vitro* differentiation and tissue formation assay

The remaining samples were stained for expression of tissue-specific markers according to standard immunofluorescence protocols, as we previously described^23^. Precisely, to confirm the identity of EPs embedded in the constructs, the antibodies rabbit monoclonal anti-cytokeratin 5 (K5) (0.077 mg/mL, 1:300 dilution, Abcom RbmAb technology) and anti-cytokeratin 14 (K14) (1.996 mg/mL, 1:300, Abcom RbmAb technology) were incubated overnight at 4 °C. To detect the presence of SGCs, samples were incubated with the antibodies mouse monoclonal anti-cytokeratin 18 (K18) (1 mg/mL, 1:300, Abcom MmAb technology) and rabbit monoclonal anti-cytokeratin 19 (K19) (1 mg/mL, 1:300, Abcom RbmAb technology). K5, K14, and K19 were incubated with red fluorophore-labeled mouse anti-rabbit secondary antibody (1:500), and K18 was inducbated with red fluorophore-labeled rabbit anti-mouse secondary antibody (1:500) for 2-hr dark incubation at room temperature. Finally, Dapi Fluoromount-G (20 mL, SouthernBiotech) was used to stain nuclei.

To detect the glandular tissue formation, pictures were taken continuously with a fluorescence microscope (Olympus, BX51) at the different time points, the process of bud emergence and glandular branching growth within 3D constructs were recorded.

In these experiments, at least 3 samples were scored in 3 independent replicates for each group and at least 100 cells were scored for each sample. The relative fluorescence intensity was quantified using Image J.

### Statistical analysis

All data are presented as mean ± SD. Statistical analysis involved two-way ANOVA with a Bonferroni post-hoc test and Student *t* test in Graphpad Prism. Statistical significance was defined as *p < 0.05. Three independent trials were carried out unless otherwise stated.

## Additional Information

**How to cite this article**: Liu, N. *et al.* 3D bioprinting matrices with controlled pore structure and release function guide in vitro self-organization of sweat gland. *Sci. Rep.* **6**, 34410; doi: 10.1038/srep34410 (2016).

## References

[b1] Fu X. B., Sun T. Z., Li X. K. & Sheng Z. Y. Morphological and distribution characteristics of sweat glands in hypertrophic scar and their possible effects on sweat gland regeneration. Chin. Med. J (Engl). 118, 186–191 (2005). 15740645

[b2] Lu C. P., Polak L., Rocha A. S., Pasolli H. A., Chen S. C. *et al.* Identification of stem cell populations in sweat glands and ducts reveals roles in homeostasis and wound repair. Cell. 150, 136–150 (2012). 22770217 10.1016/j.cell.2012.04.045 PMC3423199

[b3] Ferlin K. M., Prendergast M. E., Miller M. L., Kaplan D. S. & Fisher J. P. Influence of 3D printed porous architecture on mesenchymal stem cell enrichment and differentiation. Acta Biomater. 16, 161–169 (2016). 26773464 10.1016/j.actbio.2016.01.007

[b4] Lee C. H., Rodeo S. A., Fortier L. A., Lu C., Erisken *et al.* Protein-releasing polymeric scaffolds induce fibrochondrocytic differentiation of endogenous cells for knee meniscus regeneration in sheep. Sci. Transl. Med. 6, 266ra171 (2014). 10.1126/scitranslmed.3009696 PMC4546837 25504882

[b5] Huang S., Yao B., Xie J. & Fu X. 3D bioprinted extracellular matrix mimics facilitate directed differentiation of epithelial progenitors for sweat gland regeneration. Acta Biomater. 15, 170–177 (2015). 26747979 10.1016/j.actbio.2015.12.039

[b6] Kim K., Dean D., Wallace J., Breithaupt R., Mikos A. G. *et al.* The influence of stereolithographic scaffold architecture and composition on osteogenic signal expression with rat bone marrow stromal cells. Biomaterials. 32, 3750–3763 (2011). 21396709 10.1016/j.biomaterials.2011.01.016 PMC3075725

[b7] Duarte Campos D. F., Blaeser A., Korsten A., Neuss S., Jäkel J. *et al.* The stiffness and structure of three-dimensional printed hydrogels direct the differentiation of mesenchymal stromal cells toward adipogenic and osteogenic lineages. Tissue Engineering Part A. 21, 740–756 (2014). 25236338 10.1089/ten.TEA.2014.0231

[b8] Inzana J. A., Olvera D., Fuller S. M., Kelly J. P., Graeve O. A. *et al.* 3D printing of composite calcium phosphate and collagen scaffolds for bone regeneration. Biomaterials. 35, 4026–4034 (2014). 24529628 10.1016/j.biomaterials.2014.01.064 PMC4065717

[b9] Young S., Wong M., Tabata Y. & Mikos A. G. Gelatin as a delivery vehicle for the controlled release of bioactive molecules. J Control Release. 109, 256–274 (2005). 16266768 10.1016/j.jconrel.2005.09.023

[b10] Huang S. & Fu X. Naturally derived materials-based cell and drug delivery systems in skin regeneration. J Control Release. 142, 149–159 (2010). 19850093 10.1016/j.jconrel.2009.10.018

[b11] Hori K., Sotozono C., Hamuro J., Yamasaki K., Kimura Y. *et al.* Controlled-release of epidermal growth factor from cationized gelatin hydrogel enhances corneal epithelial wound healing. J Control Release. 118, 169–176 (2007). 17289206 10.1016/j.jconrel.2006.12.011

[b12] Ferraris C., Chevalier G., Favier B., Jahoda C. A. & Dhouailly D. Adult corneal epithelium basal cells possess the capacity to activate epidermal, pilosebaceous and sweat gland genetic programs in response to embryonic dermal stimuli. Development. 127, 5487–5495 (2000). 11076768 10.1242/dev.127.24.5487

[b13] Oshimori N. & Fuchs E. Paracrine TGF-β signaling counterbalances BMP-mediated repression in hair follicle stem cell activation. Cell Stem Cell. 10, 63–75 (2012). 22226356 10.1016/j.stem.2011.11.005 PMC3349223

[b14] Plikus M., Wang W. P., Liu J., Wang X., Jiang T. X. *et al.* Morpho-regulation of ectodermal organs: integument pathology and phenotypic variations in K14-Noggin engineered mice through modulation of bone morphogenic protein pathway. Am. J. Pathol. 164, 1099–1114 (2004). 14982863 10.1016/S0002-9440(10)63197-5 PMC1614723

[b15] Leung Y., Kandyba E., Chen Y. B., Ruffins S. & Kobielak K. Label retaining cells (LRCs) with myoepithelial characteristic from the proximal acinar region define stem cells in the sweat gland. PLoS One. 8, e74174 (2013). 24058524 10.1371/journal.pone.0074174 PMC3776797

[b16] Pati F., Jang J., Ha D. H., Kim S. W., Rhie J. W. *et al.* Printing three-dimensional tissue analogues with decellularized extracellular matrix bioink. Nat. Commun. 5, 3935 (2014). 24887553 10.1038/ncomms4935 PMC4059935

[b17] Fan L., Du Y., Huang R., Wang Q., Wang X. *et al.* Preparation and characterization of alginate/gelatin blend fibers, J. Appl. Pol. Sci. 96, 1625–1629 (2005).

[b18] Poldervaart M. T., Gremmels H., van Deventer K., Fledderus J. O., Oner F. C. *et al.* Prolonged presence of VEGF promotes vascularization in 3D bioprinted scaffolds with defined architecture. J. Control Release. 184, 58–66 (2014). 24727077 10.1016/j.jconrel.2014.04.007

[b19] Xiao X., Wang W., Liu D., Zhang H. Q., Gao P. *et al.* The promotion of angiogenesis induced by three-dimensional porous beta-tricalcium phosphate scaffold with different interconnection sizes via activation of PI3K/Akt pathways. Sci Rep. 5, 9409 (2015). 25797242 10.1038/srep09409 PMC4369742

[b20] Huebsch N. Lippens E., Lee K., Mehta M., Koshy S. T. *et al.* Matrix elasticity of void-forming hydrogels controls transplanted-stem-cell-mediated bone formation. Nat Mater. 14, 1269–1277 (2015). 26366848 10.1038/nmat4407 PMC4654683

[b21] Chaudhuri O., Gu L., Klumpers D., Darnell M., Bencherif S. A. *et al.* Hydrogels with tunable stress relaxation regulate stem cell fate and activity. Nat Mater. 15, 326–334 (2016). 26618884 10.1038/nmat4489 PMC4767627

[b22] Caiazzo M., Okawa Y., Ranga A., Piersigilli A., Tabata Y. *et al.* Defined three-dimensional microenvironments boost induction of pluripotency. Nat Mater. 15, 344–352 (2016). 26752655 10.1038/nmat4536

[b23] Xie J. F., Yao B., Han Y. T., Shang T., Gao D. Y. *et al.* Cytokeratin Expression at Different Stages in Sweat Gland Development of C57BL/6J Mice. Int J Low Extrem Wounds. 14365–371 (2015). 10.1177/1534734615611563 26680749

